# Classical fragile-X phenotype in a female infant disclosed by comprehensive genomic studies

**DOI:** 10.1186/s12881-018-0589-6

**Published:** 2018-05-10

**Authors:** Paula Jorge, Elsa Garcia, Ana Gonçalves, Isabel Marques, Nuno Maia, Bárbara Rodrigues, Helena Santos, Jacinta Fonseca, Gabriela Soares, Cecília Correia, Margarida Reis-Lima, Vincenzo Cirigliano, Rosário Santos

**Affiliations:** 1 Centro de Genética Médica Jacinto de Magalhães (CGMJM), Centro Hospitalar do Porto, CHP, E.P.E., Praça Pedro Nunes, 88 4099-028 Porto, Portugal; 2 Unit for Multidisciplinary Research in Biomedicine, Abel Salazar Institute of Biomedical Sciences, University of Porto – UMIB-ICBAS-UP, Porto, Portugal; 3 GDPN - Labco diagnostics, Synlab Group, Genética e Diagnóstico Pré-natal, Porto, Portugal; 4 Unidade de genética molecular, Centro de Genética Médica Jacinto de Magalhães, Centro Hospitalar do Porto, CHP, E.P.E, Porto, Portugal; 5 Mestranda Biologia Molecular e Celular Universidade de Aveiro, Unidade de Genética Molecular, Centro de Genética Médica Jacinto de Magalhães, Centro Hospitalar do Porto, CHP, E.P.E, Porto, Portugal; 6 Unidade de Neurociências da criança e adolescente, Serviço de Pediatria, Centro Hospitalar de Vila Nova de Gaia/Espinho (C.H.V.N.Gaia/Espinho), E.P.E, Vila Nova de Gaia, Portugal; 7 Unidade de genética médica, Centro de Genética Médica Jacinto de Magalhães, Centro Hospitalar do Porto, CHP, E.P.E, Porto, Portugal; 8 GDPN- Labco diagnostics, Synlab Group, Genética e Diagnóstico Pré-natal, Porto, Portugal; 9 Department of Molecular Genetics, Labco diagnostics, Synlab Group, Esplugues de Llobregat, Barcelona, Spain; 10 UCIBIO/REQUIMTE, Departamento de Ciências Biológicas, Faculdade de Farmácia, Universidade do Porto, Porto, Portugal

**Keywords:** Developmental disabilities in females, *FMR1* methylated full mutation, Fragile-X syndrome, Skewing of X-chromosome inactivation, Xq28 deletion

## Abstract

**Background:**

We describe a female infant with Fragile-X syndrome, with a fully expanded *FMR1* allele and preferential inactivation of the homologous X-chromosome carrying a de novo deletion. This unusual and rare case demonstrates the importance of a detailed genomic approach, the absence of which could be misguiding, and calls for reflection on the current clinical and diagnostic workup for developmental disabilities.

**Case presentation:**

We present a female infant, referred for genetic testing due to psychomotor developmental delay without specific dysmorphic features or relevant family history. *FMR1* mutation screening revealed a methylated full mutation and a normal but inactive *FMR1* allele, which led to further investigation. Complete skewing of X-chromosome inactivation towards the paternally-inherited normal-sized *FMR1* allele was found. No pathogenic variants were identified in the *XIST* promoter. Microarray analysis revealed a 439 kb deletion at Xq28, in a region known to be associated with extreme skewing of X-chromosome inactivation.

**Conclusions:**

Overall results enable us to conclude that the developmental delay is the cumulative result of a methylated *FMR1* full mutation on the active X-chromosome and the inactivation of the other homologue carrying the de novo 439 kb deletion. Our findings should be taken into consideration in future guidelines for the diagnostic workup on the diagnosis of intellectual disabilities, particularly in female infant cases.

## Background

Fragile-X syndrome (FXS, MIM #300624) is the most common cause of hereditary intellectual disability (ID) with an X-linked inheritance pattern and incomplete penetrance in females. FXS has been shown to be caused by an unstable CGG repeat within the 5’untranslated region of the fragile mental retardation-1 (*FMR1*) gene [1, 2]. This repeat is highly polymorphic with normal alleles harbouring 8 to 54 CGGs, while full expansions have more than 200 repeats [3]. The expansion within the full mutation range usually accompanied by abnormal methylation of the *FMR1* gene promoter and repetitive region, reducing Fragile X mental retardation protein (FMRP) expression [4]. The physical, neurocognitive and behavioural FXS features are therefore the result of a typical loss-of-function mutation with epigenetic changes (histone modifications and DNA methylation), by mechanisms still not entirely understood [5]. A recent FXS epidemiologic study estimates the frequency of affected males at 1.4:10,000 and that of affected females at 0.9:10,000 [6]. The typical FXS phenotypic characteristics have been described in males. Around 50% of full mutation female carriers present some degree of cognitive impairment (from mild learning disability to severe cognitive dysfunction), but usually less severe than in FXS males [7, 8]. FXS should be considered in the presence of particular physical characteristics such as long face, large and protruding ears and macroorchidism, combined with ID or autistic behaviour. Besides familial cases, both males and females with ID, even those without the other clinical signs, should be tested for fragile-X because the pathognomonic FXS features are not always obvious or present [9]. Herein, we present the case of a female infant, referred for genetic consultation due to developmental delay and hyperactivity, without specific dysmorphic features or relevant family history. *FMR1* mutation screening revealed the presence of a methylated full mutation and a normal but inactive allele, which prompted further investigation.

## Case presentation

**Fig. 1:**
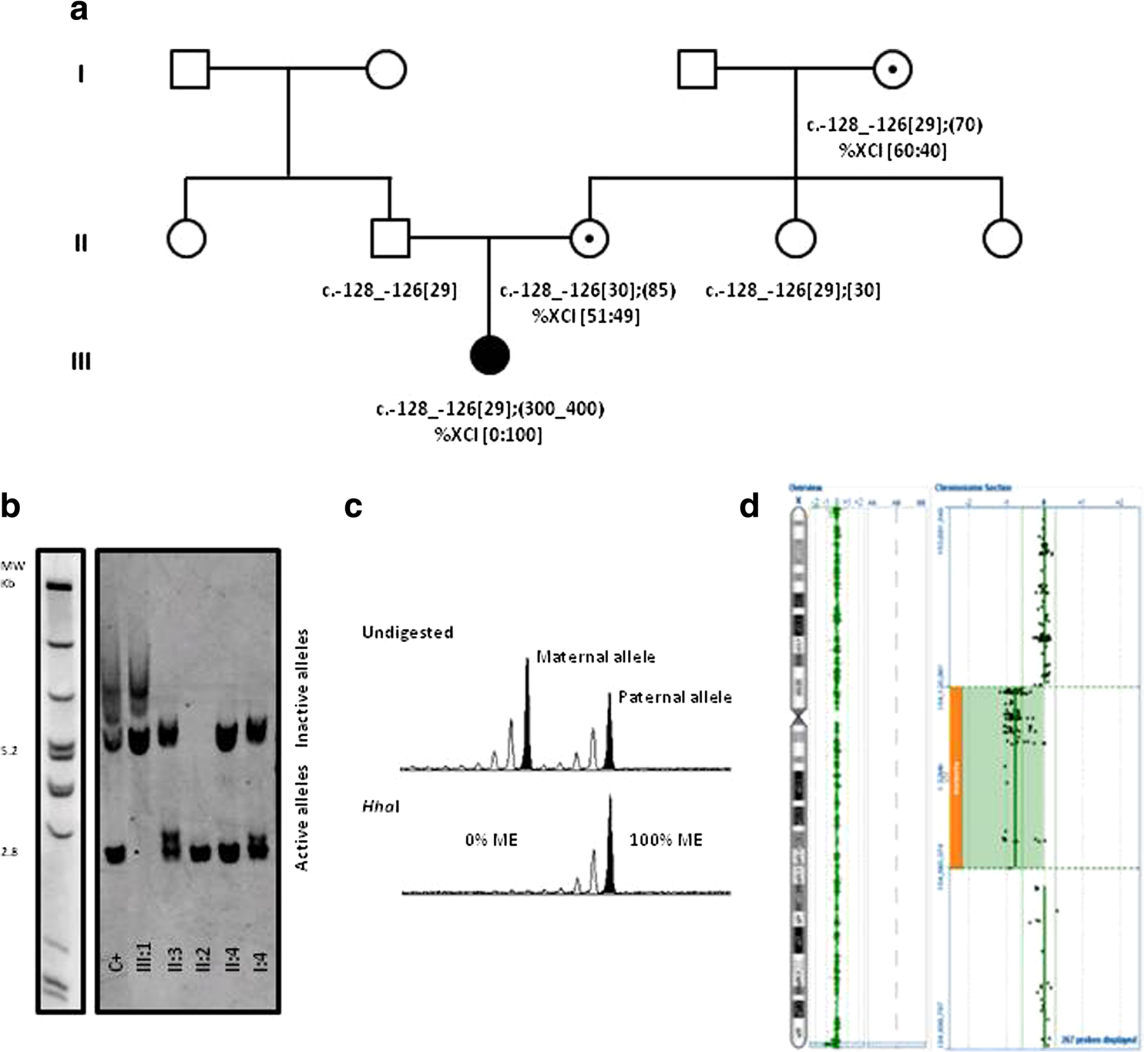
Pedigree and summary of overall laboratory findings. **a** – Proband’s pedigree (III:1) showing *FMR1* genotyping and XCI ratio results. **b** – Southern blot analysis using GLFXDig1 probe (Gene Link^TM^ , Hawthorne, NY, US) and DNA Molecular Weight Markers II and III, DIG-labeled from Merck KGaA, Darmstadt, Germany; Female control with a full mutation (C+) c.-128_-126[30];[250_400]; Proband III:1 showing complete absence of normal, active *FMR1* allele. Premutation carriers (II:3 and II:4) show four fragments corresponding to the normal active, expanded active (2.8 kb and above) and normal inactive and expanded inactive (5.2 kb and above) alleles [21]. **c** - HUMARA results obtained in proband’s leukocytes (III:1) [10]. *Hha*I completely digested the maternal allele indicating that the other allele is fully methylated (ME) and suggesting total skewing of the XCI pattern. The additional two females tested (II:3 and I:4) showed a normal XCI pattern (data not shown). **d** – Array Comparative Genomic Hybridisation (aCGH) performed using the Cytochip ISCA 8x60K (Cambridge Bluegnome, Illumina Inc., San Diego CA,USA) showed a deletion of 439Kb in chromosome X within band Xq28, classified as pathogenic, involving the genes: *F8*; CTD-2183H9.7; *EEF1A1P31*; CTD- 2183H9.3; *FUNDC2*; *CMC4*; *MTCP1*; *BRCC3*; RP11-143H17.1; *VBP1*; RP13-228 J13.9; *RAB39B*; *CLIC2*; RP13-228 J13.6; RP13-228 J13.10. Array design included a median resolution of 120Kb throughout the genome (Backbone) with increased density oligonucleotide probes in selected regions associated with clinically relevant phenotypes, in line with the International Standard Cytogenetic Array (ISCA) consortium. Analysis was performed using the Bluefuse software (Cambridge Bluegnome, Illumina Inc., San Diego CA, US) coupled with publicly available databases. Mutation nomenclature guidelines suggested by the Human Genome Variation Society (HGVS) (http://varnomen.hgvs.org/) were used. *FMR1* reference sequence NM_002024.5

The proband was first referred to our genetics clinic at 11 months of age. She showed developmental delay and hyperactivity without specific dysmorphic features and with irrelevant family history. At 33 months the developmental profile was similarly delayed with limited speech and language acquisition. The proband had early intervention for speech and language as well as occupational therapy. The last evaluation at 44 months revealed remarkable inattentiveness besides persistence of developmental delay, poor language skills and a global developmental profile equivalent to 30 months and handling skills equivalent to 24 months. Irrelevant dysmorphisms included redundant eyelids, bulbous nose and protruding ears. Analytic studies showed slightly elevated creatine phosphokinase levels and normal creatinine metabolism. The parents and other relatives gave informed consent for samples to be used in this research study, approved by the medical ethics committee of the Centro Hospitalar do Porto (CHP, E.P.E.). Following the proband’s referral for genetic testing, a normal karyotype was found together with an *FMR1* full mutation and a normal but inactive allele (Fig. 1). Co-segregation studies identified two at-risk females with *FMR1* premutations and excluded *FMR1* expansion in a maternal aunt. HUMARA testing [10], carried out on the proband’s peripheral blood, showed complete skewing of the X-chromosome inactivation (XCI) pattern. Further *FMR1* analysis, by AmplideX® *FMR1* mPCR, showed absence of size and/or methylation mosaicism (above 1%) and confirmed that the normal-sized allele was inactivated, suggesting that another cause was implicated in the skewing (Fig. 1) [11]. A good candidate for skewed XCI is *XIST* (MIM 314670), a non-protein coding gene, as a C to G transversion present in the minimal promoter (position − 43) underlies skewing in some families [12]. Several authors recognized that a C to A transversion at the same position results in skewing of XCI towards the active X homologue of heterozygous females [12, 13], while others found no such association [14]. XCI skewing in the proband was further investigated by sequencing the *XIST* promoter. No pathogenic variants were identified.

aCGH analysis was performed, revealing a 439 kb deletion in Xq28 (chrX:154,120,961–154,560,374 (hg19)) encompassing 16 genes. Similar deletions have been shown to be associated with extreme deviation of XCI, compatible with the skewing observed in our case [15, 16].

## Discussion and conclusions

Diagnostic yield for chromosomal microarray analysis (CMA) in unexplained ID is between 15%- 20%, half of these carrying a de novo copy number variant [17]. CMA to assess DNA copy number is currently recommended as a first-tier test for postnatal evaluation of patients with developmental delay, intellectual disability, autism spectrum disorders and/or multiple congenital anomalies [17]. In this case, however, application of CMA technology in the first instance, revealing a de novo 439 kb deletion, could have misguided the diagnostic workup; for example, searching for hemizygous point mutations in the *RAB39B* and *CLIC2* genes – included in this recurrently duplicated/deleted region – both of which have been implicated in ID [18, 19]. Although the deletion breakpoints were not sequenced in our case, according to previous publications, one can assume that they are within the directly orientated low-copy repeat (LCR) regions int22h-1 and int22h-2, located in the *F8* gene (MIM #300841) [15]. There is no family history of haemophilia A, although the observed preferential XCI could explain the absence of haemophilic clinical signs in the proband. Another deleted gene in this region is *VBP1* (MIM #300133), heterozygous deletions of which associate with high miscarriage rates in females without cognitive function involvement [16]. A methylated *FMR1* full mutation was identified and according to Godler et al.*,* the presence of an expansion is closely associated with an X-inactivation pattern skewed towards the mutated chromosome [20]. Here, we have showed that the developmental delay is the cumulative result of a methylated *FMR1* full mutation on the active X-chromosome and the inactivation of the other homologue carrying the de novo Xq28 deletion, although we were unable to exclude *FMR1* tissue mosaicism or the presence of other X-linked recessive pathogenic variants in genes involved in the Xq28 deletion. Overall, this report describes an atypical fragile-X female infant whose phenotype one may speculate should develop in a similar manner as that described for typical FXS males. This case poses additional challenges for genetic counseling and also calls for reflection on the clinical and diagnostic workup for developmental disabilities, particularly in infant females: a positive aCGH result should not hinder *FMR1* sizing and methylation analysis and vice-versa. In the present case, first-tier aCGH could have misguided the clinical geneticist towards sequencing several genes involved in ID (e.g. *RAB39B* and *CLIC2*) resulting in a completely distinct diagnostic workflow.

## Abbreviations

aCGH: Array Comparative Genomic Hybridisation
CMA: Chromosomal microarray analysis
*FMR1*: Fragile mental retardation-1 gene
*FMRP*: Fragile mental retardation protein
*FXS*: Fragile-X syndrome
ID: Intellectual disability
ISCA: International Standard Cytogenetic Array
LCR: Low-copy repeat
XCI: X-chromosome inactivation
